# “We are toothless and hanging, but optimistic”: sub county managers’ experiences of rapid devolution in coastal Kenya

**DOI:** 10.1186/s12939-017-0607-x

**Published:** 2017-09-15

**Authors:** Mary M. Nyikuri, Benjamin Tsofa, Philip Okoth, Edwine W. Barasa, Sassy Molyneux

**Affiliations:** 1 Health Systems and Research Ethics group. KEMRI-Wellcome Trust Research Program, P.O Box 230, 80108 Kilifi, Kenya; 2 Health Economics Research Unit, KEMRI-Wellcome Trust Research Programme, P.O Box 43640–00100, Nairobi, Kenya; 3 The Ethox Centre, Department of Public Health, University of Oxford, Old Road Campus, Headington, Oxford, OX3 7LF UK; 4 The Centre for Clinical Vaccinology and Tropical Medicine, Nuffield Department of Medicine, University of Oxford, Old Road Campus, Headington, Oxford, OX3 7LF UK; 5 Strathmore University Business School, Ole Sangale Road, Madaraka, P.O. Box 59857–00200, Nairobi, Kenya; 6 Nuffield department of Medicine, University of Oxford, Oxford, UK

**Keywords:** The District/county, Organisation change, Decentralization, Devolution, Mid level managers, Health systems, Policy implementation, Kenya

## Abstract

**Background:**

In March 2013, Kenya transitioned from a centralized to a devolved system of governance. Within the health sector, this entailed the transfer of service provision functions to 47 newly formed semi-autonomous counties, while policy and regulatory functions were retained at the national level. The devolution process was rapid rather than progressive.

**Methods:**

We conducted qualitative research within one county to examine the early experiences of devolution in the health sector. We specifically focused on the experience of change from the perspective of sub-county managers, who form the link between county level managers and health facility managers. We collected data by observing a diverse range of management meetings, support supervision visits and outreach activities involving sub-county managers between May 2013 and June 2015, conducting informal interviews wherever we could. Informal observations and interviews were supplemented by fifteen tape recorded in depth interviews with purposively selected sub-county managers from three sub-counties.

**Results:**

We found that sub county managers as with many other health system actors were anxious about and ill-prepared for the unexpectedly rapid devolution of health functions to the newly created county government. They experienced loss of autonomy and resources in addition to confused lines of accountability within the health system. However, they harnessed individual, team and stakeholder resources to maintain their jobs, and continued to play a central role in supporting peripheral facility managers to cope with change.

**Conclusions:**

Our study illustrates the importance in accelerated devolution contexts for: 1) mid-level managers to adopt new ways of working and engagement with higher and lower levels in the system; 2) clear lines of communication during reforms to these actors and 3) anticipating and managing the effect of change on intangible software issues such as trust and motivation. More broadly, we show the value of examining organisational change from the perspective of key actors within the system, and highlight the importance in times of rapid change of drawing upon and working with those already in the system. These actors have valuable tacit knowledge, but tapping into and building on this knowledge to enable positive response in times of health system shocks requires greater attention to sustained software capacity building within the health system.

## Background

### District health managers in decentralised health systems

In Low and Middle Income Countries (LMICs), health system reform has followed a global trend towards decentralization of services from central governments and large hospitals to local governments and district health clinics [15]. This involves the transfer of decision-making from the central governmental body, to local officials in order to tailor health care to the needs of local populations and increase access to medicines and treatments across all regions of a nation [4]. Decentralization has been promoted as a reform that will improve health system performance through improved efficiency, responsiveness and local accountability [5]. The district remains the backbone of decentralized health systems in most LMICs. Existing within a hierarchical structure, it is the basic level responsible for operational tasks such as support supervision and plays an integrative role of interfacing between national-level policy formulation, and ensuring policy implementation at facility level [6, 33].

Health systems are people centred, strongly shaped by the decisions and actions of historical and current day actors [2, 9], and district managers are key actors in district health systems. District managers typically have planning and coordination roles, including guiding, mentoring and overseeing staff operating at sub-district and facility levels. Depending on the level of dentralisation, they are also responsible for strategic planning and oversight of the health system in their districts [33], for translating policies from higher levels of health system hierarchies to district level, and are answerable in turn to actors back up the system.

Despite the recognition that people ultimately determine the character and performance of the health system, few studies have examined district health managers’ experiences in their roles, particularly in the face of significant organisation change [42]. Organisation change in this context refers to change in governance structures and roles, calling for new ways of engaging, problem solving and common understanding [12, 42] A recent study conducted by colleagues in South Africa highlighted that district managers can play important and very particular roles as change intermediaries during times of change. Positioned at the interface between more senior managers and front line employees, their interpretation of change can be influential not only on their own perspectives and behaviours, but also if and how they help others through change, including through how they ‘make sense’ of change upwards to senior managers, laterally with peers and downwards to those who report to them; i.e., their ‘sense-making’ roles [19].

This paper describes how district managers experienced and interpreted this change within a context of a rapidly devolving health system in Kenya. In March 2013, Kenya transitioned from a centralized to a devolved system of governance. Devolution is one of the four forms of decentralization described by Anne Mills et al. as the shifting of power from central to peripheral institutions [29]. Devolution is a relatively extensive form of decentralization in any setting, and in the health sector in Kenya entailed the complete transfer of service provision functions from central level to 47 newly formed semi-autonomous counties. Policy and regulatory functions were retained at the national level. We share district managers’ experience of devolution from inside the health system, describing change as it unfolded over time for them. We hope by sharing these experiences to generate insights into the importance of involving the institutions and actors charged with managing change in times of politically driven reforms. Although addressed in a particular setting, the experiences presented in this paper may have relevance in a range of other health system settings undergoing decentralisation [16]. It is has been argued that decentralisaton should be a means to ensuring good governance processes such as accountability, rather than an end in itself [5]. Therefore more broadly, drawing on governance ideas from the policy implementation literature, the paper contributes to the still limited literature on the important role mid level managers play in organization change and more specifically in times of politically driven health sector reforms in LMICs [20, 43].

### Kenya’s health sector under devolution

In 2013, after a national election that ushered in a new government, Kenya transitioned into a devolved system of government with a central government and 47 semi-autonomous units called counties [45]. To facilitate the transition, a Transition Authority was formed with the responsibility of overseeing the transfer of devolved functions through policy and legal advice, resource mobilization, oversight, capacity building and coordination over a three year period [24]. Within the health sector, devolution entailed the transfer of health service delivery functions to county governments. County pharmacies and health facilities, ambulance services, and primary health care promotion were transferred to county health systems, while National government retained health policy and regulatory functions (Ministry of Health 2013a; [23]). In addition, the two Ministries of Health (Ministry of Medical Services (MOMS) and Ministry of Public Health and Sanitation (MOPHS)) that existed under the previous governance arrangement were merged into one Ministry of Health (MOH) [45].

**Fig. 1:**
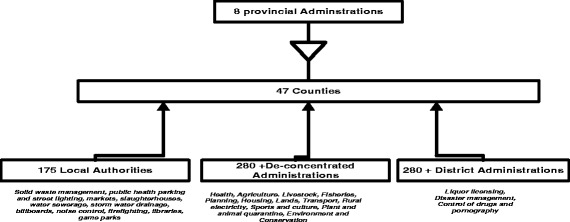
How counties were formed from provinces, districts and municipalities [23]

**Fig. 2:**
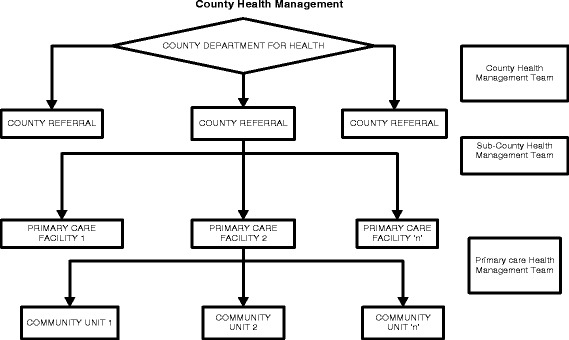
The District as sub county unit within the county system. *Source*: [34]

It was envisioned that at the county level, county departments of health would be formed to provide an enabling institutional and management structure responsible for “coordinating and managing the delivery of healthcare mandates and services at the county level”. In addition, county health management teams were to provide “professional and technical management structures” in coordinating the delivery of health services through available health facilities (Kenya Health Policy 2012–2030). However, the envisioned three year transition period was cut short soon after the March 2013 election, when there was rapid transfer of functions to governments, the old districts became known as sub-counties, and in many cases the district health management teams (DHMTs) became known as sub-county health management teams (SCHMTs). Figures 1 and 2 shows the changes in governance in the country. Counties were formed by merging 175 local authorities, former districts, and district level departmental structures. The former 8 provinces were also collapsed to pave way for the counties.

Kenya’s devolution has been described by the World Bank as “one of the most ambitious implemented globally” [18] because, besides the creation of 47 new counties, the process has also involved the creation of new systems of administration that have absorbed some or all of three prior systems of administration as shown above.

The previous District Health Management Team (DHMT) under devolution therefore became a SCHMT now at a level below the County Health Management Team (CHMT), with the latter being a newly created layer under devolution. In this paper, we examine the experience of mid-level managers who were DHMT members before devolution, who transitioned to being SCHMT members. We illustrate the challenges that these managers faced at a time of significant change in health system structures and processes, and illustrate some of their coping strategies which could be argued to demonstrate resilience.

## Methods

### The ‘learning site’ approach

The data presented in this paper are part of a wider study aimed at understanding governance changes under devolution in Kenya, conducted under the umbrella of the ‘learning site’ approach that has been described elsewhere by the authors [31]. Briefly, a learning site is a long term process of collaborative research in which health managers and researchers decide together over a relatively long-term collaboration what the key health system questions and interventions are. The approach includes repeated interactions between researchers and managers over long periods of time that built trust and familiarity, and that enable the invaluable tacit knowledge of health managers to be accessed [37]. The Kenyan learning site is situated in a county on the Kenyan Coast, which consists of three sub county management teams. The county is divided into six administrative sub-counties, with these sub-counties being paired for health administration. Total number of SCHMTs by end of 2015 was three [10].

### Data collection

Data were collected primarily through non participant observations (including informal interactions and interviews) and formal in-depth interviews. MN and PO participated in a diverse range of meetings and activities over a period of more than one year soon after devolution (May 2013 and June 2015). These included management meetings, support supervision and outreach activities in all the 3 sub-counties. Towards the end of this period, fifteen tape recorded in-depth interviews with purposively selected sub-county managers from three sub-counties were carried out (Table 1). Each interview took between 45–60 min. Non participant observations began during rapid devolution and post devolution. Interviews were conducted with members of the former DHMT who transitioned into SCHMT, timed to be able to formally ask about and explore observation and informal interview data in more depth. All of these data were supplemented by observations and interviews conducted as part of the wider learning site work described above; notably for this paper, interviews with health centre and dispensary in-charges. These findings were shared with the sub county and county managers in mid 2016 as part of a half day discussion, which facilitated further collection on and reflection about the data.

**Table 1:** Sub county managers interviewed

Sub county manager	Acronym	Number interviewed
Sub county medical Officer of Health or designee	SCMOH	2
Sub county Public Health Nurse	SCPHN	3
Sub county Health Records and Information Officer	SCHRIO	2
Sub county Administrator	SCA	3
Sub county clinical officer	SCCO	1
Sub county Public Health Officer	SCPHO	3
Sub county facility management nurse	SCFMN	1
Total		15

### Data analysis

Observation notes and data from the 15 interviews were transcribed verbatim and managed using NVivo 10. The number of transcribed interviews were assumed adequate as published in other studies [13, 22]. A grounded approach to coding was used, whereby all data were reviewed and codes iteratively introduced without prior identification of potential themes. This approach was selected to enable managers’ experience of devolution as it happened/unfolded to be shared. Our findings are organised around the key themes that emerged from the data. In the discussion we then explored these emerging themes in greater depth, drawing in particular on the organisational change literature that we found supported our interpretation and deeper understanding of the data: literature on the role of mid-level managers in times of organisational change, including their sense-making roles [19], and literature on health worker productivity and motivation, and in particular the importance of the software elements of complex health systems (i.e., knowledge, skills, decision-making processes, relationships, communication practices, values and norms, as opposed to hardware elements such as infrastructure, technology and resource levels) [14].

In this paper we have selected quotes that best illustrate some of the key themes in the findings.

Ethical clearance for the study was obtained from the authors’ institution (KEMRI SSC: 2205) and from the London School of Hygiene and Tropical Medicine 6250 . Information about the project was given to all participants at county and sub county levels prior to data collection. Members of the researchers explained their role at the start of each meeting to ensure that their health service colleagues were satisfied with their presence and had an opportunity to ask any questions. Written informed consent was obtained prior to all formal interviews. Careful attention was paid to ensuring that participants’ anonymity was maintained in the documentation and write up of the study.

## Results

Clear emerging themes over the course of our analysis were the lack of preparedness of sub county managers for the changes that were coming under devolution, and the inadequate clarity for all in role and structural changes, including in finance systems and structures. We discuss these themes followed by an overview of new ways of working adopted by sub-county managers, and their reported implications.

### Lack of preparedness for and clarity about anticipated changes

The plan for health sector devolution to be implemented over a carefully planned three year period was overtaken by wider political developments and there was a rapid transfer of functions within the first few months of having a new government in March 2013 ([8].). There was therefore little time at all levels to plan and communicate with local levels about changes in the health sector. The rapid implementation of devolution caught the sub county managers unprepared for change contributing to significant anxiety and confusion among this cadre. The managers feared for the loss of their roles within the county health system, and ultimately of their jobs. This concern had already been building up. It was observed that in the meetings pre and post the 2013 elections with their old line managers at the provincial level (Fig. 1), they often requested for information about the expected new structures and their roles within them, but responses were limited to vague announcements. Managers from one sub county complained during one of these meetings that they were suffering significant ‘stress’ as a result of the uncertainty in the impending devolution process and the need for counselling. Such worries were echoed across the three sub counties during interviews:

… *We are all in a greyish zone. We don’t understand ourselves as DHMT, though we are still around, each one of us should be told where we will go*… sub county A, manager 2

… *You see as at now there is a lot of uncertainty because we actually don’t know what is ahead, there is some kind of lack of knowing, and some darkness currently is ahead…* sub county B, manager 1

In addition to lack of clarity from the provinces on roles and structures prior to devolution, once devolution began there was inadequate communication between the central and local levels on the unfolding situation and process. Often the first source of information among sub-county managers was reported to be the national or local media:

*…If you wait for the county to inform you of any changes, you are wasting your time, just read the papers*… sub county A, manager 1

Communication that was happening across both of these stages – pre and post devolution – sometimes fuelled anxieties and confusion. For example we observed some senior managers explaining that anticipated changes were likely to include loss of titles, massive transfer of human resources and even retrenchment; comments potentially linked to the lack of certainty among those senior managers on their own future positions as well. Sub-county managers described the unfolding processes as essentially top down, with limited information and involvement, despite their interest and efforts to hear and contribute more. Many sub-county managers described this situation as having significantly undermined their motivation.

*…We have not held any organised meeting, but we try and hijack any moment at seminars to voice our challenges with our managers*….Sub county C, manager 3

…*By the way it [our motivation] has gone down. It’s not the way it used to be, because the people who come are the ones we issue the licence if you don’t come nobody has the morale of making follow up…*sub county C, manager 4.

### Evolving structures and job roles

The composition of the DHMT changed as members were transferred out of the sub counties to other sub counties or to the newly established county health team as shown in Table 2 below on promotion basis. However, to maintain same number of sub county managers, former deputies or assistants to the transferred managers were promoted to full managerial status. In the process, titles were lost as the DHMT members took on a more subordinate role of sub county managers.

**Table 2:** Transfers of managers

Sub county	DHMT members who transitioned into SCHMT	DHMT members who joined the CHMT
A	8	2 Promoted to another county
	1 Promoted to county level but declined	4 Promoted to county level (accepted)
B	14	1 promoted to county level
C	13	1 promoted to another county
		1 promoted to County level

There were no terms of reference for both the CHMT and SCHMT teams from the national level, or organogram provided. A letter from the national government to the interim county managers was an attempt to restructure the DHMT, but it was not clear in this document how this was to be done or even whether that was a decision that should be made nationally or at the county level. Consequently, the directive was ignored by senior managers in the then newly forming CHMT, perhaps to give themselves more time to make sense of the rapidly changing and confusing situation.

…*there is a prescription from the top about those who are required to be in the SCHMT but then it was seen not to be working. [Our senior manager] said we should just work the way we were working so that one was nullified…*sub county A, manager 3

The new county systems that did begin to evolve did not have clearly defined structures, processes, guidelines or role clarity. There was also no clear organogram for the county health department, and even some debate about whether the emerging CHMT level was valid at all. The above process contributed to the emerging two levels of management struggling to coordinate with each other, and to there being unclear roles and lines of accountability between the SCHMTs and the CHMT.

…*To start with, I can say that there should be a proper well organized organogram because now there is just confusion; a proper organogram that will guide us on who you should report to. Currently, whatever is there is frustrating, you can do a report you copy to one person the other person calls you needs the same report, you inform that person ok I sent the report to so and so and he insists you have to resend it to me. So there is not that linkage, there is no team work. They [the CHMT members] are working as individual people which is not good*… Sub county B manager 4

Confusion and duplication in roles reportedly resulted in inefficiencies with some of those operating and the new CHMT level having little to do.

*…I don’t think they are very busy, one thing, they are very many and, for example a whole manager sitting for the whole month just waiting for a report from the sub county? To me that person is just being wasted.* …Sub county C manager 1

*… when you go there, now you find everyone is on computer but I am not used to such. They are just watching Bob Marley, looking at Facebook, not me…* sub county A manager 4

### Poorly defined financing systems and processes

The lack of an organogram and duplication of roles was exacerbated by lack of systems for financial allocation to lower level institutions, including SCHMTS, hospitals, health centres and dispensaries. Once rapid devolution took place, all funds allocated to the county health department were held at the county treasury. A challenge was that the county treasury - which had been in existence prior to devolution - lacked technical capacity to handle additional financial requests from the newly created structures as well as the previous ones. In addition, there were no mechanisms for allocating funds to the sub county management unit as there was no legal framework for it to be an accounting unit. Consequently, although the CHMT were able to access funds, the sub county managers were unable to access any funds to perform their activities.

All the sub county managers interviewed complained of lack of funds and shared their frustration in trying to access funds for implementing activities from their county line managers. Their line managers did advise them to make proposals, but many of these were reportedly not acted upon at all, or were not paid in their entirety. Some managers put it this way;

…*The County is giving us money but too little money…* sub county B manager 2

*… we used to get what we call HSSF being allocated and the District Medical Officer of Health (DMOH) had some allocation but when the counties came that allocation is not there,…so actually we have gotten challenges since the new system came in the county government. Because we can’t access funds directly, we have to request from our county then that time our county said we don’t have any money allocated; the systems have not been set in place…*sub county B manager 6

The challenges with financial flow remain a challenge at the time of writing, more than three years post devolution, contributing to significant delays in funds and associated activities, as discussed next.

*… when we were working direct with the national government we were getting immediate response, if you say we need this we get immediate response but now from the county there is some confusion, you might send a request for something then it is delayed…* sub county B manager 3

### Mid-level managers coping strategies in a changing system

#### Reducing activities

Poorly defined structures, roles and financing systems and processes had consequences for sub-county managers’ ability to carry out some of their previous core activities. A first change was to reduce the number of meetings they held. The private practitioners meeting - a forum that DHMTs had used to convey Ministry guidelines and updates to the private sector - ceased.

… *We don’t meet because we are not supported on the same, so when they come they demand at least something small because they have left their area of work from morning to that particular time..*.sub county B manager 5

The quarterly full DHMT meetings which were used to review the performance of the sub counties in terms of indicators were also inactive over the course of field work in all the three sub counties. Reasons given were the lack of finances, an unstable office of the Sub County Medical Officer of Health (SCMOH) in two sub counties and a lack of morale. Consequently the managers were unable to look at their performance as a sub county and discuss the challenges.

A meeting that used to bring all the health related stakeholders in the district, the District Stakeholders Forum was only held if there was an emergency such as an outbreak. This meeting was used before devolution for planning of health programs across the entire district, and to share resources and identify gaps in service in the community. Described as a very important forum for sharing with stakeholders their work plans, progress and pooling resources, its’ irregularity was considered detrimental to the community, to managers and to service users. The managers’ lack of oversight and voice over what health stakeholders were now doing led to worries about duplication of efforts as well as marginalisation of the most needy.

A second and related change resulting from the on-going structural changes was to how managers conducted their activities. Supportive supervision reportedly became much more irregular across all three sub counties. Managers said they used to visit facilities at least once in a quarter to support frontline workers, including by listening to their technical and social challenges.

… *We give them ideas wherever they are stuck or maybe there are nice things they have done we congratulate them … we sit with them and we correct them, it’s just an On Job Training (OJT) but also use the forum to identify gaps in skills so we can recommend training, that is where you find some people are being called for training*… sub county B manager 2

Considered by the managers as one of the core activities for SCHMT, their inability to conduct supportive supervision was perceived by themselves and by health workers to indicate the managers’ powerlessness. Interviews with frontline health workers over the same period indicated that these health workers looked forward to the supportive visits from their supervisors, and were particularly eager to interact with them at a time of such change.

#### Reorganising work routines and building on experience and relationships

In the sub county managers’ efforts to continue to conduct their roles, they appeared to have a strong sense that their duty of delivering services to the sick should not be compromised by this period of heightened change and confusion. They also had hopes that this period of uncertainty would end; an expectation boosted by a meeting they all had with one senior county official who was keen to consult with them on their challenges and possible solutions. This meeting led SCHMT members to feel that a clear organogram and legislation would emerge over time to restore their relatively independent status.

*… Am really yearning for that organogram for the county to be released…I think things will be working better…* sub county C manager 2

*…we are optimistic that legislation, the way we have been promised from this year, if at all the legislatures can pass …. the SCMOH [will get] some budget as well as what we used to get… from the hospitals. You know when such money comes it is very easy for the SCMOH to budget for its activities…*Sub county A manager 3

In this context, they harnessed individual, team and stakeholder resources to maintain their jobs, and to continue to try to play a critical role in supporting peripheral facility managers. At an individual level, managers dipped into their own pockets – with the expectation of repayment as soon as was possible from their line managers - to meet essential costs to discharge their roles, including payment of transport, mobile phone use, and internet access.

*…we are working with our pockets. You can see this [points at phone] is my personal effects, I use my modem; I have to buy my own credit to do the work of the government…* sub county C manager 3

A strong and rich team spirit was visible among many of the sub-county managers, health facility managers, frontline clinicians, and community representatives. This spirit was related in part to a long history of working together and coping with the constant mini crises of health service delivery in the county, in some cases of several decades. They therefore had created strong working and social relationships with the community and other actors in the health system, with a common sentiment being:

…. *these are my nurses, my doctors, my people (the community), so I cannot let them down, we have to help each other…*Sub county A manager 2

A sense of team spirit was demonstrated among the sub-county managers in the way they organised their work schedules and shared their resources. For example, in the absence of performance appraisals from their senior managers, they used weekly meetings to check on each other’s activities and achievements (as well raise worries and concerns, and problem solve together). Also, they creatively divided out tasks; streamlining different activities in one facility for example:

*…Sometimes we might find me as a SCHMT we don’t have money but a certain program like HIV/AIDS might have some money so you might find one person from that department will go and see what is happening then you will also check on other small things in the facility and where there are issues they will be reported…*Sub county A manager 3

The strong networks between sub-county managers, and between managers and other health actors in the county, supported their creativity. For example, to cope with the funding challenges, sub-county managers relied on funding from other vertical programmes who agreed “to help each other out”. Sub-county managers also leveraged funds from donors and non-government organizations that had been working in the county to support activities:

…*so the partners are on and off, but we consolidate their support and that from private sector to carry out some activities such as support supervision*…..Sub county A manager 4

Due to the above outlined adaptive mechanisms, the sub-county managers continued to discharge their former DHMT responsibilities of coordinating the delivery of health services in the county. For example, they continued to appraise and review progress of service delivery through monthly meetings with facility managers in the county main town.

…*The in-charges meetings are there every month, they are important as various issues are discussed and we try with the help of partners to conduct them without fail…..we identify facilities that have challenges, so we ca follow up during supervision…* Sub county C manager 2

They also continued to carry out supportive supervision of facilities by focusing on facilities that were facing challenges ranging from lack of reporting, through conflict among cadres working at the facility, to a failure by the in charge to attend monthly in charge meetings.

*….We have a schedule for facilities we need to visit including the private facilities but we had backlog because of the challenge of transport so we now conduct supervision two days in a week, so we have been scheduling for Tuesday and Wednesday for the supervision unless there is any challenge…..*sub county C manager 4*.*

NGOs also proved to be a useful resource for helping managers cope with technical tasks. For example in the face of a cholera outbreak, sub-county managers were able to mobilise resources from two non-governmental organizations (NGOs) to travel to the outbreak location, collect samples and send them to the national government chemist for analysis, as well as transport suspected infected people to the county hospital for treatment. These actions were thought to have successfully contained the outbreak:

*…, we called upon the stakeholders and they really supported us. Some gave us commodities such as ORS, water treatment, others gave us vehicles for mobilization, and others provided refreshments for the teams in the field. …*Sub county A manager 4

## Discussion

In this paper, we have described how a group of managers in a county at the Coast experienced rapid change and how they mediated this change in a lower middle income country. We tracked change as it happened from within the health system, and have shown that – as is the case in other contexts [36], the political nature and rapidity of the devolution process brought significant challenges. Pre devolution, the managers in our county of focus were anxious about what lay ahead; a situation compounded by several inter-related factors, including a lack of guidelines at national level, the inevitable need in a devolved system for counties to evolve their own structures and systems, and problems in level and style of communication. Post devolution, the sudden creation of a new structure resulted in two managerial layers without a clear organogram or terms of reference. Together with continued inadequacies in communication between key actors in the health system, this contributed to role duplication, poorly defined financing systems and processes, resource access delays, and relationship challenges. Despite these challenges – some of which linger on three years later - sub county managers over the research period drew on their social networks and relationships, their desire to maintain their jobs and roles and their shared values and team spirit, to keep many essential activities going.

The important implementation role played by mid level managers in times of organisation change generally has been noted in the management literature as key to successful, less disruptive changes [38–40]. In times of health sector reform processes the role played by mid level managers in implementing change has been acknowledged by others in other contexts [18, 30, 33, 36]. For example, a World Bank study of decentralisation in Ghana noted that successful decentralisation requires that decentralized entities be prepared to take over their new responsibilities. For instance, there should be sufficient numbers of qualified personnel to handle decentralized responsibilities, staff contracting and management systems, merit-based remuneration, and to allow for corruption control [11].

In our case, sub county managers had to adapt to the unexpectedly rapid changes by adopting new ways of working. The experience of these managers under rapid devolution was similar to South East Asian countries who implemented rapid decentralization without adequate preparation and capacity building [44]. Literature on a decade of decentralization of health care services in Java, indicates that the district had limited capacity to assume the new roles in the decentralization arrangements. The situation was further complicated by lack of regulations to support the main decentralization legislation [44]. In our context, sub-county managers in their adaptations began to demonstrate a form of managing change that has been called ‘unlearning of yesterday’s ideas’ [17, 32]. In the face of financial limitations and lack of role clarity, they reorganised and rethought their working structures and routines: they had to discontinue some meetings that needed financial facilitation and began utilising other meetings to achieve the same programme goals. These findings contrast with some literature from organisation change which indicates that it can be hard for people to abandon their usual beliefs and procedures that have arisen from previous successful experiences [41]. In our county the ability to unlearn was linked in part to their strong relationships with each other and with other key actors, and their optimism that the situation would soon improve. Of interest will be to observe over time whether some elements of these new ways of working have introduced some efficiencies that can continue even as resources are made available, or whether these changes are ‘undone’ or ‘unlearned’ again at that point [26, 27].

We have reported elsewhere how much the facility managers – health workers in charge of health centres and dispensaries - appreciated the support of these managers over this time of rapid change [31]. In effect the sub county managers were, for these facility in-charges, playing the role of interpreting and translating complex and worrying changes in such a way as to sustain as far as possible motivation and every day practice at the micro level [38, 39]. By maintaining contact with the frontline workers through irregular support supervision and regular facility in-charges meetings in a central place, sub-county managers were able to communicate with and support in-charges, and effectively cushion them from some of the challenges being played out at higher levels of the health system hierarchy. This observation is similar to one made in South Africa where it was shown that communication among actors at different levels of the health system can keep morale for staff high, even in the context of significant difficulty [28]. In our setting an important role of mid-level managers in their interactions with in-charges was of interpreting and communicating change [3, 19], even in the face of significant insecurities and concerns of their own. Often closer to key health system actors in the community than the more senior managers, and often with long histories of working in the area, they were able to adapt to change, by drawing on former networks to continue with their roles such as conducting disease specific health campaigns. Their own feeling of being ignored and left out of the devolution process potentially drove them further to play this sense making role; to actively seek out opportunities to reassure staff and to continue with their own role of coordinating service at the frontline. Of interest given the important role that these mid-level managers took on, was that in debates at county level prior to and over the course of devolution, the role of mid-level managers was deemphasized in comparison to that of the more senior managers. This has been described as typical in situations of planned radical change where their contributions are seen as much weaker, being viewed as part of the disinterest and barriers to change [21].

It has been shown in other contexts that mid-level managers work best when the quality of their work environment is good (Hutchinson 2002), and that productivity and motivation of health workers are linked to adequacy of resources [7, 25, 35]. Our study supports these findings in showing that a lack of financial resources led to demotivated managers. However our findings also show that in a very difficult and turbulent work environment, sub-county managers demonstrated flexibility and optimism in an effort to maintain their professional identity and performance. Thus, for example, they introduced new forms of mutual appraisal, shared resources accessed through external networks and even drew on their own personal resources. In doing this we note the importance of intrinsic motivation and commitment to maintain a professional identity, as well as team work and optimism about a better future, as key. These findings complement a study conducted in Uganda where health workers in a decentralised system worked hard to maintain a professional identity after the health sector reforms weakened their positions as professionals [25]. Our findings also suggest that through these activities, sub-county managers were able to build their own and facility managers’ feelings of being a team, contributing to some positivity and mutual accountability in at a very turbulent and insecure time [19]. Drawing on the distinction made by Ortiz Aragon and others between three interacting dimensions of organisational capacity: the hardware of infrastructure, technology and funding levels; the tangible software of knowledge, skills and processes of decision making; and the intangible software of relationships, communication practices, values and norms, our sub-county managers were drawing upon and feeding into the software elements of what was is a highly complex health system. It has been argued elsewhere that the intangible features of capacity can be particularly important in shaping the behaviours of those working in an organisation and underpinning organisations’ “power to perform” [14] We certainly noted the importance of these intangible elements of sub-county managers’ roles. Anticipating and managing the effect of change on crucial software elements of health systems therefore has the potential to support service delivery in Kenya and elsewhere in the short and longer terms [1].

### Study limitations

The findings in this paper are based on data collected only in one county and under a situation of continuous and on-going change although informal discussions suggest issues remain relevant today. This study employed a qualitative design, and therefore the findings on the importance of SCHMTs in addition to CHMTs need to be carefully interpreted as they are based on observations and views including from CHMT members and facility managers, as well as SCHMTs themselves; but not able to look into cost and inefficiencies of an additional layer of management. Nevertheless, the findings are relevant even in other settings and potentially useful in the context of quite drastic changes such as devolution.

## Conclusion

Sub-county health managers bridge the gap between front line health staff and higher levels of the health care system. They are tasked with duties and responsibilities that potentially have a direct positive impact on service delivery at the frontline. Rapid devolution under a new constitution was characterised first by anxiety and fear by managers due to poor communication, lack of involvement in the process, and sudden structural changes with poor role clarity; and secondly by poorly defined financing systems and processes that led to delays and inefficiencies in fund flow. This caused significant challenges for sub county managers, including loss of autonomy and resources in addition to confused lines of accountability within the health system. Nevertheless, they harnessed individual, team and stakeholder resources to maintain their jobs, and continued to play a central role in supporting frontline workers.

Our study illustrates the value of examining organisational change from the perspective of key actors within the system, and highlights the importance in accelerated devolution contexts for engagement of such ‘mid-level managers’ in managing change. Our study illustrates the importance of mid-level managers adopting new ways of working and engaging up and down the system, and of clear lines of communication during reforms to these actors. The importance of anticipating and managing the effect of change on intangible software issues such as trust and motivation is also highlighted.

In times of such massive organisation change, the process should not be divorced from the people whose tacit knowledge and unquantifiable people skills are key in driving and managing change. More broadly, greater attention needs to be paid to preparing the health system at all levels for positive responses to shocks by sustained capacity building of software elements.
